# Special Notice

**Published:** 1898-12

**Authors:** 


### SPECIAL NOTICE.

Attention is called to an advertisement upon another page. An accident pratically closed the professional career of the advertiser, and he desires to dispose of the volumes of the *Dental Cosmos* from the first issue. To those who contemplate having a private library there is nothing better as a foundation than the volumes of a dental journal.

